# Influence of graphene on the multiple metabolic pathways of *Zea mays* roots based on transcriptome analysis

**DOI:** 10.1371/journal.pone.0244856

**Published:** 2021-01-04

**Authors:** Zhiwen Chen, Jianguo Zhao, Jie Song, Shenghua Han, Yaqin Du, Yuying Qiao, Zehui Liu, Jun Qiao, Weijia Li, Jingwei Li, Haiyan Wang, Baoyan Xing, Qiliang Pan

**Affiliations:** 1 Key Laboratory of National Forest and Grass Administration for the Application of Graphene in Forestry, Institute of Carbon Materials Science, Shanxi Datong University, Datong, P.R. China; 2 School of Chemistry and Chemical Engineering, Shanxi Datong University, Datong, P.R. China; Henan Agricultural University, CHINA

## Abstract

Graphene reportedly exerts positive effects on plant root growth and development, although the corresponding molecular response mechanism remains to be elucidated. Maize seeds were randomly divided into a control and experimental group, and the roots of *Zea mays* L. seedlings were watered with different concentrations (0–100 mg/L) of graphene to explore the effects and molecular mechanism of graphene on the growth and development of *Z*. *mays* L. Upon evaluating root growth indices, 50 mg/L graphene remarkably increased total root length, root volume, and the number of root tips and forks of maize seedlings compared to those of the control group. We observed that the contents of nitrogen and potassium in rhizosphere soil increased following the 50 mg/L graphene treatment. Thereafter, we compared the transcriptome changes in *Z*. *mays* roots in response to the 50 mg/L graphene treatment. Transcriptional factor regulation, plant hormone signal transduction, nitrogen and potassium metabolism, as well as secondary metabolism in maize roots subjected to graphene treatment, exhibited significantly upregulated expression, all of which could be related to mechanisms underlying the response to graphene. Based on qPCR validations, we proposed several candidate genes that might have been affected with the graphene treatment of maize roots. The transcriptional profiles presented here provide a foundation for deciphering the mechanism underlying graphene and maize root interaction.

## Introduction

Nanomaterials have been widely used in the fields of electronics, machinery, energy, and biomedicine [1–4]. Research has indicated that certain nanomaterials may improve seed germination rate and promote plant growth in certain species [5, 6]. Nanomaterials broadly include metals, metal oxides, polymers, and carbon nanoparticles, but only carbon nanomaterials have attracted considerable attention because of their unique chemical properties [6]. Graphene is a member of the carbon nanomaterials family and is the most promising engineered nanomaterial due to its huge surface area, unparalleled mechanical properties, and electrical and thermal conductivity [7]. Notably, the large surface area can be chemically modified to harbor a variety of oxygen-containing functional groups, including carboxyl, hydroxyl, and carbonyl groups, which confer higher water dispersity to these modified graphene structures [8].

Myriad studies have evaluated the positive effects of graphene on plant growth and development. For a few species, including tomato, spinach, and wheat, graphene treatment reportedly promotes seed germination. In a study conducted using tomato, graphene-treated seeds showed accelerated germination compared to control (CK) seeds, possibly due to graphene penetrating the seed husks and consequently facilitating water uptake [9]. Likewise, He et al. reported that graphene could act as a water transporter to promote germination of spinach seeds in soil [10]; however, He et al. previously reported that hydrated graphene ribbon could promote aged seed germination in wheat [11].

Research in several species (e.g., rice, tomato, cilantro, garlic, fava bean, and maize, among others) have shown that graphene positively affects roots generally by promoting growth and/or by increasing the number of lateral roots [9, 12–15]. In several cases, this may also result in increased gibberellic acid production [9] and/or greater biomass [12–14]. In rice, graphene-treated plants exhibited increased seedling weight [12], whereas graphene treatment in cilantro and garlic promoted the growth of numerous organs, ultimately leading to elevated yield [16]. In maize, low concentrations of sulfonated graphene are associated with increased reactive oxygen species (ROS) scavenging in roots, leading to both altered root morphology and improved seedling health [17]. Other possible benefits of graphene on plant growth and development include the activation of reproduction [16], reduction of the toxic effects of drought and salt stresses [18], increased carrier potential for the slow release of fertilizer, inhibition of pathogens [19, 20], and/or improved utilization efficiency of nutrients [21–23].

To date, multiple studies have focused on the physiological and/or phenotypic response to graphene exposure, with few studies characterizing the molecular response to graphene. The goal of the present study was to explore the effects of graphene on gene expression and to characterize the molecular response to graphene exposure regarding root growth and development in *Zea mays* L. seedlings.

## Materials and methods

### Graphene characterization

Graphene was obtained and generated in our lab. The characteristics of graphene were analyzed by using ultraviolet-visible absorption spectrogram and Raman spectroscopy (HORIBA, LabRAM HR Evolution). The Raman spectra were obtained using Renishaw inVia™ Qontor with a 532-nm excitation laser. The morphology of graphene was examined using scanning electron microscopy (SEM, TESCAN MAIA 3 LMH) and transmission electron microscopy (TEM, TecnaiG2F20 S-TWIN TMP).

### Maize plant cultivation and graphene exposure treatment

Similar-sized maize seeds were divided into five groups (30 seeds in each group), germinated in potting soil in a growth chamber, and the resulting seedlings were maintained in a controlled environment at 28°C (daytime) and 20°C (night-time), with a 16-h light/8-h dark photoperiod. Graphene was diluted to five different concentrations with deionized water (0, 20, 25, 50, and 100 mg/L) and neutralized to pH 6.3–6.5 with an aqueous solution of sodium hydroxide (0.1 M). Each solution was used as a treatment for one group of maize seeds/seedlings. Each graphene treatment was applied to the group via irrigation once per week, beginning with seed planting. After germination, seedlings were watered weekly with 1 L of the working solution containing the respective concentration of graphene. After 30 days (d) of exposure to graphene, the maize roots were thoroughly washed with deionized water, dried with absorbent paper to remove surface water, flash-frozen in liquid nitrogen, and stored at -80°C until RNA extraction.

### Root architecture analysis of maize seedlings

Maize seedlings from each treatment group were used for root architecture analysis. Similar to the procedures followed for samples collected for RNA-seq, maize roots were washed and harvested after 30 d of graphene exposure. Roots were scanned using the Epson Perfection V850 Pro (Seiko Epson Corp., Tokyo, Japan) at 600 dpi, and the scanned images were subsequently analyzed by WinRHIZO (Version 4.0b, Regent Instruments Inc., Quebec, Canada) [24], which is designed to measure root-specific traits. General root architecture traits were measured by WinRHIZO, including total root length, total projection area, total surface area, root volume, number of root tips, and root forks.

### RNA extraction, library construction, and sequencing

Total RNA of maize roots from the CK and 50 mg/L graphene-treated group was extracted using the RNAprep pure plant kit (TIANGEN, Shanghai, China) according to the manufacturer’s instructions. A total of 1 μg purified mRNA was used for cDNA library construction using the NEBNext UltraTM RNA Library Prep Kit for Illumina (NEB, USA), following the manufacturer’s instructions. Briefly, mRNA was purified from total RNA using poly-T oligo-attached magnetic beads. Fragmentation was conducted using divalent cations under elevated temperature conditions using the NEBNext First Strand Synthesis Reaction Buffer (5X). First-strand cDNA was synthesized using random hexamer primer and M-MuLV Reverse Transcriptase. Second-strand cDNA synthesis was subsequently performed using DNA Polymerase I and RNase H. The remaining overhangs were converted into blunt ends via exonuclease/polymerase activities. After adenylation of the 3′ ends of DNA fragments, the NEBNext Adaptors containing hairpin loop structures were ligated to proceed with hybridization. The AMPure XP bead system (Beckman Coulter, Beverly, USA) was used to select cDNA fragments of ~300 bp. PCR amplification of the library was performed using Universal PCR primers and an Index (X) Primer with the Phusion High-Fidelity DNA polymerase. Finally, PCR products were purified (AMPure XP system) and library quality was assessed using the Agilent Bioanalyzer 2100 system. Clustering of the index-coded samples was performed using the cBot Cluster Generation System and the TruSeq PE Cluster Kit v4-cBot-HS (Illumina) according to the manufacturer’s instructions. After cluster generation, the prepared libraries were sequenced using the Illumina NovaSeq platform and 150-bp paired-end reads were generated. Three biological replicates were performed for both the CK and graphene treatment groups.

### RNA-seq data quality control and read mapping

Raw data (raw reads) were initially processed using the FASTX-Toolkit (http://hannonlab.cshl.edu/fastx_toolkit/) to remove adapter-containing reads and/or those of a low quality (including poly-N). These clean reads were then mapped to the *Z*. *mays* (assembly B73 RefGen_v4) reference genome [25] using HISAT2 [26, 27]. Only reads with a perfect match or one mismatch were retained to calculate expression. Clean data are available from the Genome Sequence Archive in the BIG Data Center of Sciences (https://bigd.big.ac.cn/) under accession number CRA002623.

### Quantification of gene expression levels and differentially expressed gene (DEG) analysis

Gene expression levels were determined as fragments per kilobase of transcript per million fragments mapped (FPKM), using the following formula:FPKM = {cDNA Fragments\over {Mapped Fragments (Millions) *Transcript Length(kb)}}.

Differential expression analysis of the two groups was performed using the DESeq2 [28]. DESeq2 provides a statistical method for calculating differential expression using a model based on the negative binomial distribution. The resulting *p*-values were adjusted using the Benjamini-Hochberg approach [29] for controlling the false discovery rate (FDR). Genes with an adjusted *p*-value < 0.01 and two-fold or greater expression change identified by DESeq2 [28] were considered differentially expressed. The Multiple Experiment Viewer (MeV) [30] was used to display the gene expression patterns.

### DEG functional annotation and enrichment analyses

DEGs were assigned functions based on the following databases: nr (NCBI non-redundant protein sequences, ftp://ftp.ncbi.nih.gov/blast/db/), nt (NCBI non-redundant nucleotide sequences, ftp://ftp.ncbi.nih.gov/blast/db/), Pfam (the database of homologous protein families, http://pfam.xfam.org/), COG (Clusters of Orthologous Groups of proteins, http://www.ncbi.nlm.nih.gov/COG/), Swiss-Prot (a manually annotated and reviewed protein sequence database, http://www.uniprot.org/), KO (KEGG Ortholog database, http://www.genome.jp/kegg/), and GO (Gene Ontology, http://www.geneontology.org/).

Gene Ontology (GO) enrichment of the DEGs was analyzed using the R package goseq, which uses a Wallenius non-central hypergeometric distribution [31] that can adjust for gene length bias of DEGs. We used KOBAS [32] to test for statistical enrichment of DEGs in KEGG pathways.

### RNA extraction, cDNA synthesis, and qRT-PCR validation

Total RNA from the CK and 50 mg/L graphene-treated group was extracted for *qRT-PCR* analyses using the RNAprep pure plant kit (TIANGEN, Shanghai, China) according to the manufacturer’s instructions. The resulting RNAs were treated with DNase I before synthesizing cDNA with oligo (dT) primers and M-MLV Reverse Transcriptase (Invitrogen); these products were diluted 5-fold before use. For quantitative real-time PCR (qRT-PCR), the Primer5 software was used to design gene-specific forward and reverse primers (S1 Table). Analyses were performed with the SYBR-Green PCR Mastermix (TaKaRa) using a Mastercycler (Mastercycler RealPlex; Eppendorf Ltd, Shanghai, China). The *Z*. *mays* GAPDH (*ZmGAPDH*) gene was used as an internal reference [33], and the relative amount of the amplified product was calculated following the 2-ΔΔCt method [34]. Relative expression levels were normalized by calibrating with the CK sample from roots.

### Measurement of N and K contents in rhizosphere soil of maize plants

The soil nutrient analyzer TPY-6PC developed by Zhejiang Tuopu Yunnong Technology, Co, Ltd, was used to determine nitrogen and potassium contents of the seedling rhizosphere soil according to the manufacturer’s instructions. The rhizosphere soil was sampled with a 5-point sampling method according to the manufacturer’s instructions. Soil samples were thoroughly mixed and dried for 24 h and passed through a 1-mm sieve before measurement.

### Statistical analysis

Each treatment was conducted in triplicate, and the results are presented as mean ± standard deviation (SD). The data were analyzed using a one-way analysis of variance (ANOVA). The significance of differences between mean values was determined using the least significant difference (LSD) test at a 0.05 probability level. Pairwise comparisons were made using the Student's *t*-test and differences were regarded as significant at *p* < 0.05. All statistical analyses were performed using SPSS 21 (Predictive Analytics Software statistics 21).

## Results

### Characterization of graphene

**Fig 1:**
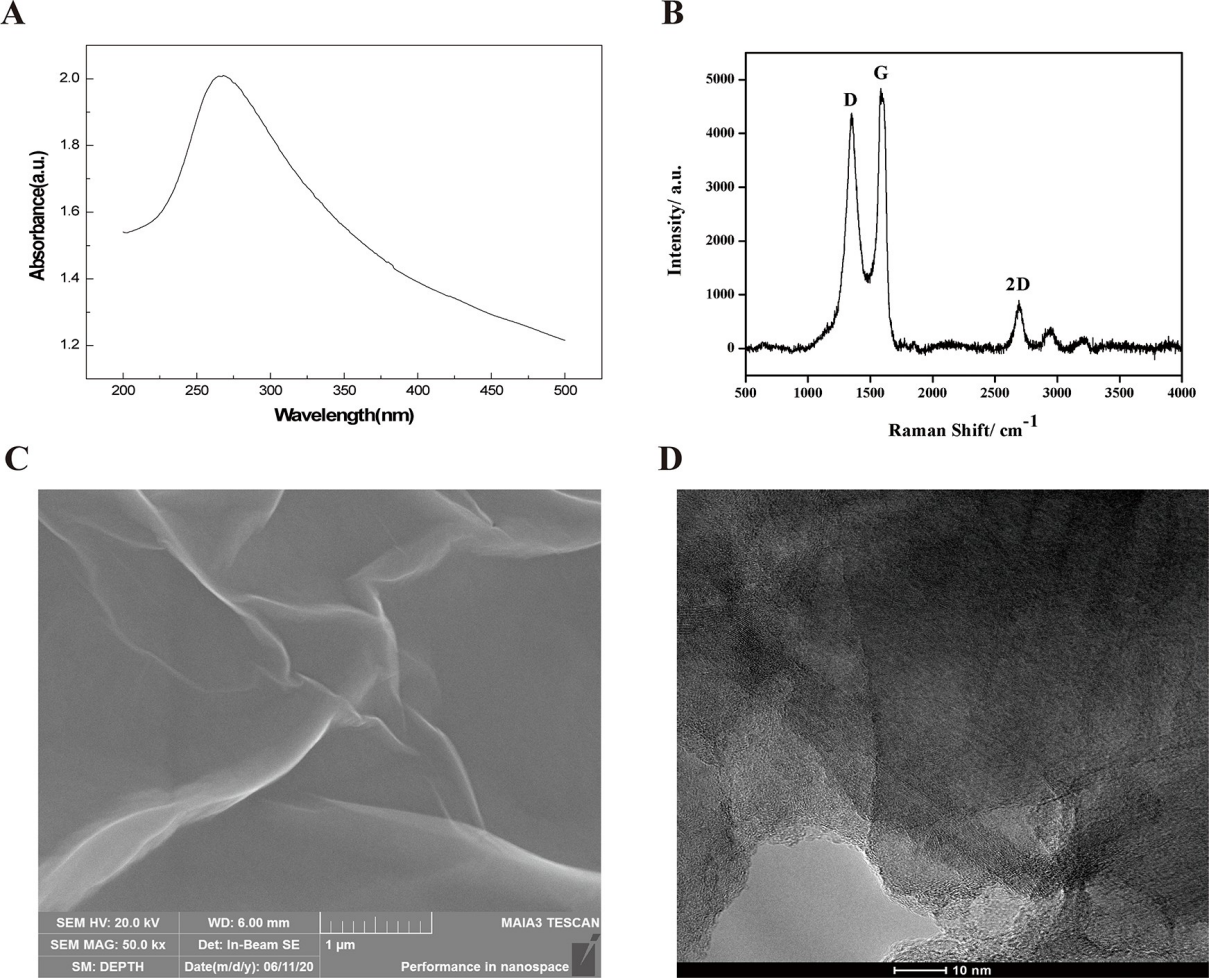
Characterization of graphene. (A) Ultraviolet-visible absorption spectrogram. (B) Raman spectra. (C) Transmission electron microscopy (TEM) image. (D) TEM image.

The ultraviolet-visible absorption spectrogram (Fig 1A) showed a remarkable absorption peak at 270 nm, a typical peak for graphene. Raman spectroscopy (Fig 1B) showed that the G peak appeared near 1576 cm^-1^, which is generated by the stretching and movement of sp2 hybridized atoms in carbon rings or long chains, representing the ordered sp2 bond structure. Peak D appeared near 1348 cm^-1^, which is indicative of a sp3 hybridized structure, representing defects and amorphous structures at the edges of the graphene. A wide 2D peak appeared near 2707 cm^-1^, indicating that the number of graphene layers prepared was within 10 layers. High-resolution scanning electron microscopy (Fig 1C) and TEM (Fig 1D) analyses indicated that the graphene was present in a transparent sheet structure with a slightly wrinkled and undulated surface. The number of graphene layers prepared was 3–5, as observed by TEM.

### Exogenous graphene promotes the growth and development of maize roots

**Fig 2:**
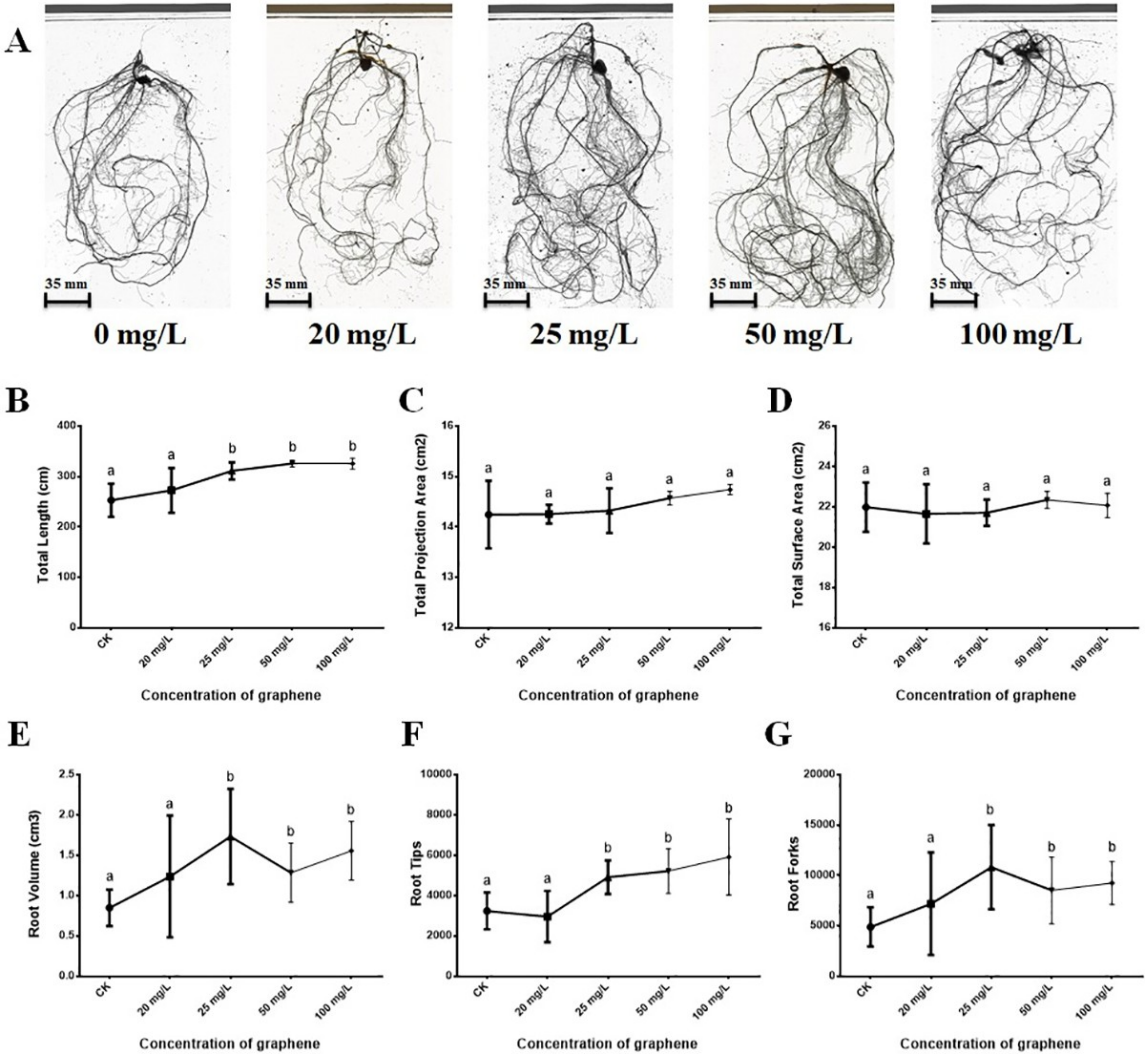
Root architecture analysis of maize seedlings in response to five concentrations of graphene (0, 20, 25, 50, and 100 mg/L). (A) Root morphology in response to five concentrations of graphene. (B) Graphene effects on the total root length of maize seedlings. (C) Graphene effects on the total projection area of maize seedlings. (D) Graphene effects on the total surface area of maize seedlings. (E) Graphene effects on the root volume of maize seedlings. (F) Graphene effects on the number of root tips of maize seedlings. (G) Graphene effects on the number of root forks of maize seedlings.

The maize seedling roots were treated with five concentration gradients of graphene and a CK. At the seedling stage, the phenotypes of maize plants were observed (S1 Fig), indicating that 50 mg/L graphene treatment significantly promoted the growth of maize plants. We next focused on the root architecture of maize seedlings in response to the CK and five concentration gradients of graphene was investigated. Root growth and development of maize seedlings in response to graphene treatments were promoted, especially at the 50 mg/L graphene concentration (Fig 2A). Root architecture traits, including total root length, total projection area, total surface area, root volume, and the number of root tips and root forks were measured (Fig 2B–2G). Compared to the CK group, 25, 50, and 100 mg/L graphene treatments increased the total root length (Fig 2B), root volume (Fig 2E), and the number of root tips (Fig 2F) and root forks (Fig 2G) of maize seedlings. Total projection area (Fig 2C) and total surface area (Fig 2D) were not affected by any graphene treatment. For those traits affected by graphene, those treated with 50 mg/L graphene showed significantly higher values than those exhibited by the CK (Fig 2). Based on the above-mentioned assay results, the 50 mg/L graphene concentration was used for the subsequent experiments and analyses.

### Transcriptome sequencing

To gain insights into the mechanisms by which graphene induces a promoting effect leading to enhanced root development, a comparative transcriptome analysis was performed. We collected root tissue samples from maize seedlings treated with 50 mg/L graphene (X100) and used untreated seedlings as the corresponding CKs. All samples were used for transcriptome sequencing with three biological replicates (S2 Table). In total, 170.02 million raw reads were obtained for the CK libraries (CK-1, CK-2, and CK-3), and 166.69 million raw reads were obtained for the X100 libraries (X100-1, X100-2, and X100-3). After removing adapter-containing and low-quality sequences along with contaminated reads, 24.15 Gb and 23.92 Gb high-quality clean bases were obtained from the CK and X100 libraries, respectively (S2 Table). Using the *Z*. *mays* genome B73 [35], the number of mapped clean reads was 43.67–60.13 million for the CK libraries (79.65%–87.30% mapped; 77.66%–85.09% uniquely mapped) and 49.56–59.40 million for the X100 libraries (77.83–%84.46% mapped; 75.66%–82.18% uniquely mapped) (S3 Table).

### DEGs in *Z*. *mays* roots in response to graphene treatment

We first calculated the Pearson correlation coefficient (PCC) for all genes and generated a heatmap plot showing changes in gene expression (as shown in S2A Fig). The correlation coefficients of the three biological replicates were greater than 0.90, indicating that the RNA-seq data were reliable for further analysis. Based on principal component analysis of six samples, the transcriptional response observed in *Z*. *mays* roots exposed to 50 mg/L graphene and CK treatments exhibited two levels of gene expression (as shown in S2B Fig).

We were specifically interested in the identification of transcripts that were differentially expressed in the root samples in response to graphene treatment, indicative of genes that might be related to root development in response to graphene treatment. The expression value of each gene was calculated using fragments per kilobase of transcript per million fragments mapped (FPKM). A two-fold change and a *p*-value of less than 0.05 were set as the cutoffs to define genes with significant differential expression (S2C Fig). We identified 962 DEGs, among which 792 were graphene-induced and 170 were graphene-repressed (S2D Fig).

### Gene enrichment analysis for DEGs

**Fig 3:**
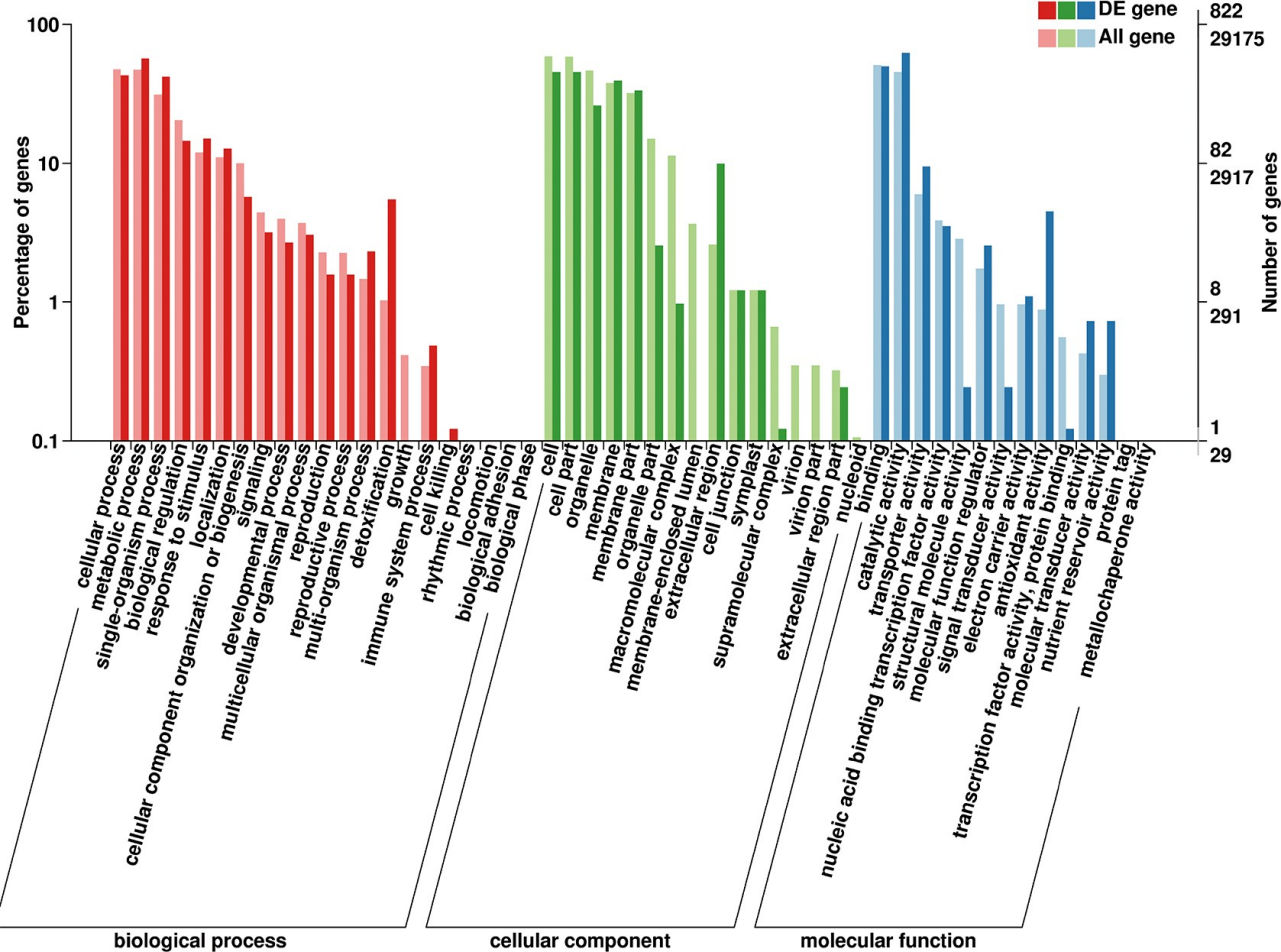
Gene ontology (GO) enrichment analysis of differentially expressed genes (DEGs) after exposure to graphene. The X-axis represents the biological functions (molecular function, biological process, and cellular component) of these DEGs. The Y-axis represents the percentage or number of genes categorized into different functional pathways.

To investigate possible biological functions that determined the different responses of the maize plants to 50 mg/L graphene treatment, we used GOseq [31] to perform GO category enrichment analysis for DEGs. Fig 3 lists the results of the GO analysis for DEGs after graphene treatment. GO terms associated with important biological processes were enriched in maize exposed to graphene treatment, including cellular, metabolic, developmental, and immune system processes, biological regulation, response to stimulus, and detoxification. Cellular components, such as cell, membrane, and organelle parts were also enriched. Molecular function enrichment consisted of catalytic activity, transporter activity, nucleic acid-binding transcription factor activity, antioxidant activity, and transcription factor activity.

DEGs were referenced against the COG database [36] to classify gene function and homology (results in S3 Fig). Most DEGs were found in orthologous groups related to secondary metabolites biosynthesis, transport and catabolism, carbohydrate transport and metabolism, amino acid transport and metabolism, lipid transport and metabolism, and defense mechanisms.

**Fig 4:**
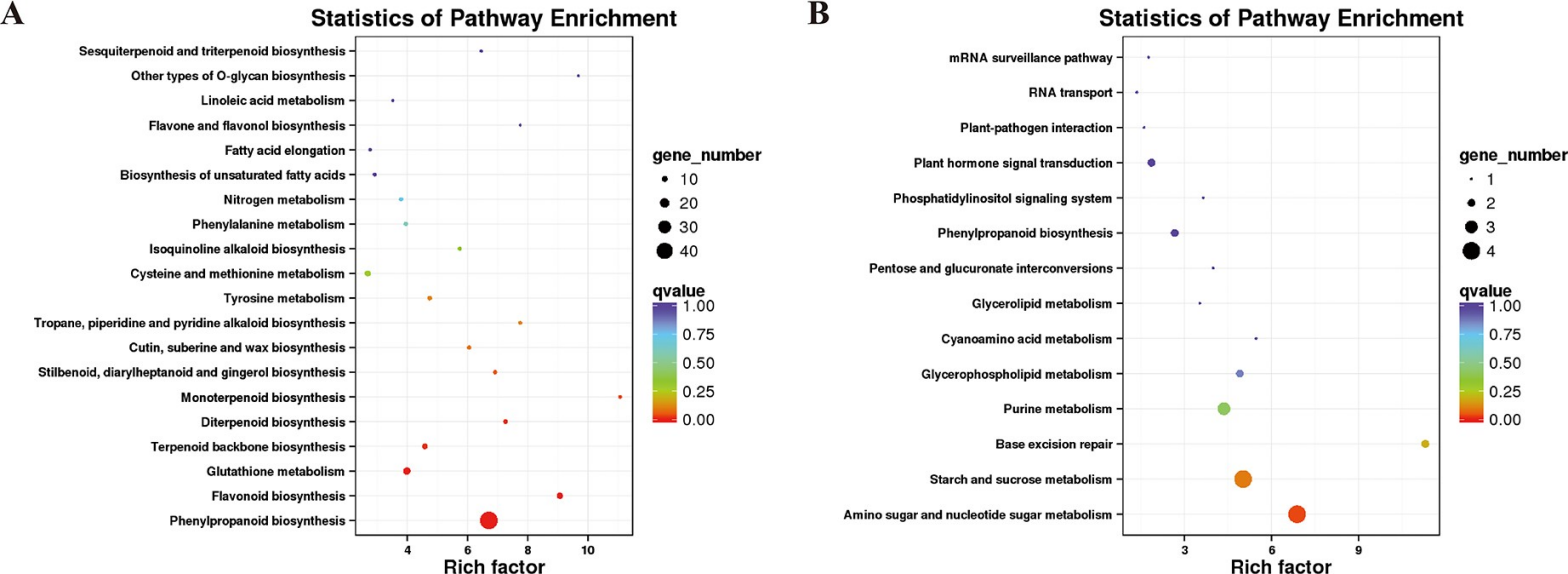
KEGG pathway analysis of enriched differentially expressed genes. (A) Top 20 pathways of significantly upregulated genes (left). (B) Top 14 pathways of significantly downregulated (right) genes.

DEGs were also subjected to KEGG pathway analysis to further inform their functional categorization. S4 Fig lists the results of the KEGG analysis for DEGs. Most DEGs were categorized belonging to functional pathways responsible for the following: 1) metabolism, including phenylpropanoid biosynthesis, glutathione metabolism, flavonoid biosynthesis, carbon metabolism, amino sugar and nucleotide sugar metabolism, cysteine and methionine metabolism, terpenoid backbone biosynthesis, biosynthesis of amino acids, as well as starch and sucrose metabolism; 2) cellular process of peroxisomes; and 3) environmental information processing, including plant hormone signal transduction, ABC transporters, phosphatidylinositol signaling system, circadian rhythm in plants, and plant-pathogen interactions. Those DEGs exhibiting expression upregulation were assigned to 73 KEGG pathways, including phenylpropanoid biosynthesis, glutathione metabolism, flavonoid biosynthesis, and nitrogen metabolism (Fig 4A). Conversely, DEGs with downregulation of expression were significantly enriched in only 14 KEGG pathways, including amino sugar and nucleotide sugar metabolism, starch and sucrose metabolism pathways, as well as plant hormone signal transduction (Fig 4B). The results reveal that graphene may affect the expression of maize root genes, resulting in upregulated expression of a majority of genes. The enrichment analysis illustrated that graphene treatment exerted extensive and distinct effects on the life processes in maize.

### Transcription factors enriched in maize plants exposed to graphene

We found that the GO term “transcription factor activity, protein binding” was significantly enriched in maize roots subjected to graphene treatment (Fig 3). Transcription factors are DNA-binding proteins that play a key role in gene transcription and expression and which mediate many processes. Many transcription factors in the roots of maize responded to graphene treatment, and the responses differed with respect to upregulation or downregulation (Table 1). Forty-four maize transcription factor genes, classified into seven different families according to PlantTFDB [37], were differentially expressed in response to the graphene treatment, including ERF, WRKY, bHLH, MYB and MYB-like, NAC, AP2, and MADS-box. Among these, 32 transcription factor genes were upregulated and 12 were downregulated. The transcription factor genes activated in *Z*. *mays* roots in response to the graphene treatment mostly belonged to the MYB and MYB-like, WRKY, NAC, and bHLH families, suggesting that these transcription factor genes might respond specifically to graphene in *Z*. *mays* roots.

**Table 1:** Differentially expressed transcription factor (TF) genes between the control (CK) and graphene treatment of root samples in *Zea mays*.

TF family	Gene Numbers	Up	Down
ERF	4	3	1
WRKY	9	8	1
bHLH	6	5	1
MYB and MYB-like	12	10	2
NAC	7	3	4
AP2	4	3	1
MADS-box	2	0	2
total	44	32	12

**Fig 5:**
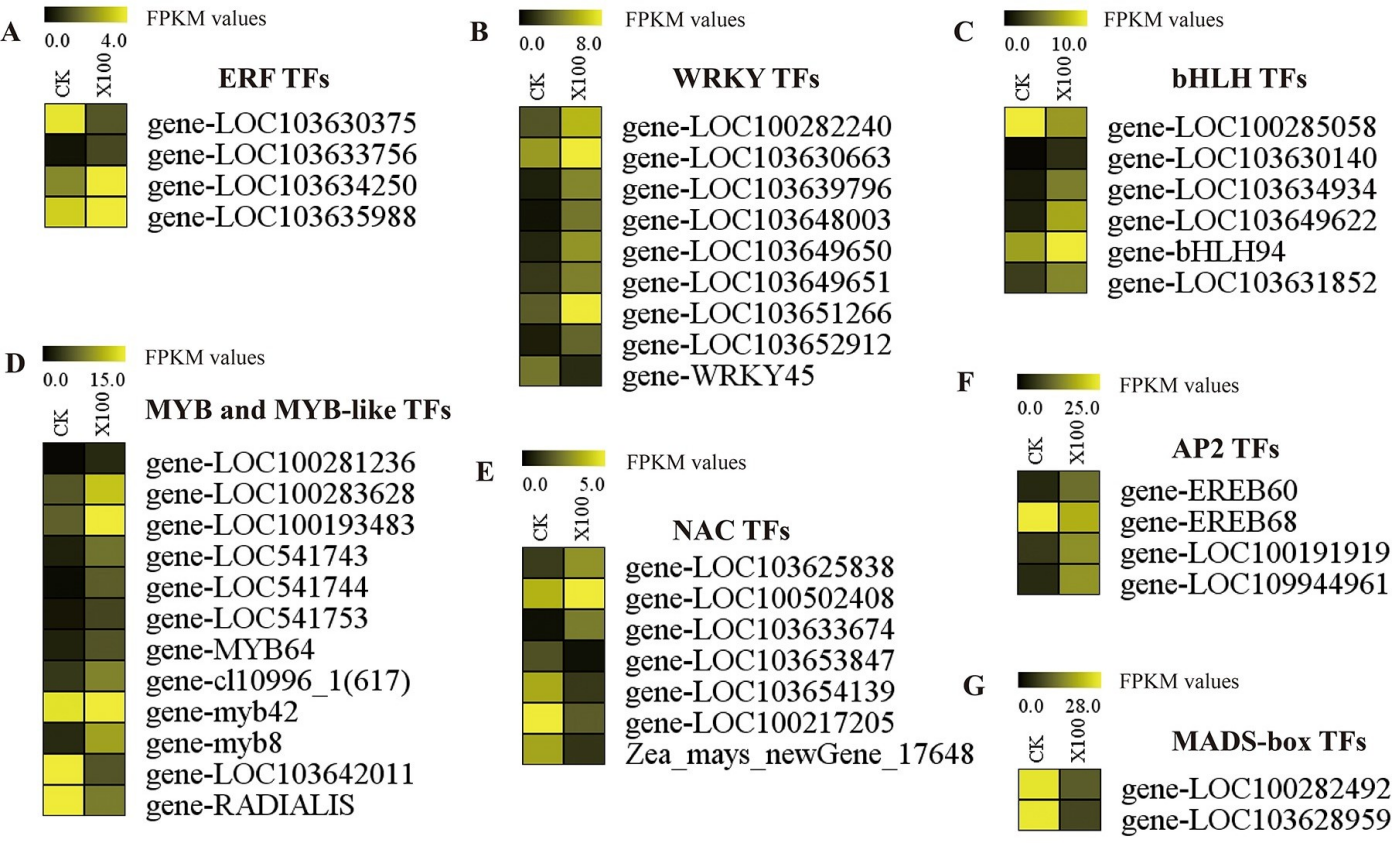
Differentially expressed transcription factor (TF) genes between the control (CK) and graphene treatment of root samples in *Zea mays*. The colored bars represent the FPKM values of the DEGs. (A) ERF. (B) WRKY. (C) bHLH. (D) MYB and MYB-like. (E) NAC. (F) AP2. (G) MADS-box.

Studies have shown that plant root development can be regulated by ERF [38], WRKY [39, 40], bHLH [41–43], MYB, and MYB-like [44–47], NAC [48, 49], AP2 [50, 51], and MADS-box [52, 53] transcription factor (TF) genes. After exposure of *Z*. *mays* roots to graphene treatment, there were three upregulated and one downregulated ethylene-responsive (ERF) transcription factor (TF) genes (Fig 5A), eight upregulated and one downregulated WRKY TF genes (Fig 5B), five upregulated and one downregulated bHLH TF genes (Fig 5C), ten upregulated and two downregulated MYB and MYB-like TF genes (Fig 5D), three upregulated and four downregulated NAC TF genes (Fig 5E), three upregulated and one downregulated AP2 TF genes (Fig 5F), and two downregulated MADS-box TF genes (Fig 5G).

As mentioned above, the total root length, root volume, and the number of root tips and root forks (Fig 2) of maize seedlings were increased after 50 mg/L graphene treatment. These seedlings exhibited improved root phenotypes that could be affected by those differentially expressed transcription factor genes. Therefore, these TFs were considered as candidate graphene-responsive genes and could be considered internal factors which promote the development of roots in *Z*. *mays*.

### Plant hormone signaling pathway

**Fig 6:**
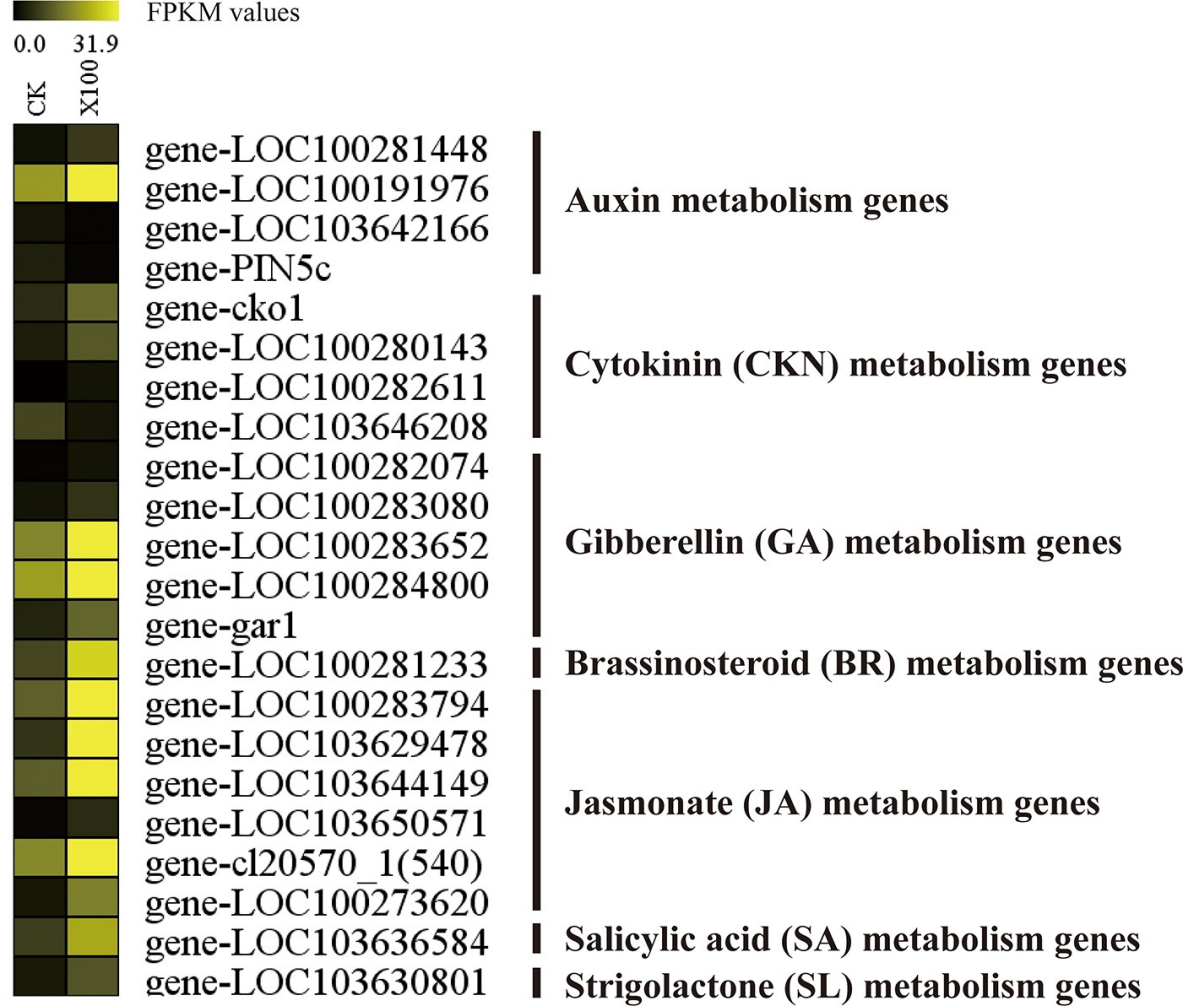
Twenty-two plant hormone signaling pathway genes are differentially expressed. Differentially expressed genes (DEGs), including four auxin metabolism genes, four cytokinin (CKN) metabolism genes, five gibberellin (GA) metabolism genes, one brassinosteroid (BR) metabolism gene, six jasmonate (JA) metabolism genes, one salicylic acid (SA) metabolism gene, and one strigolactone (SL) metabolism gene.

Plant hormones, including auxins, cytokinin (CKN), gibberellin (GA), abscisic acid (ABA), ethylene, brassinosteroid (BR), jasmonate (JA), salicylic acid (SA), and strigolactone (SL) play critical roles in plant processes, including growth, development, and adaptation to external changing environments [54–57]. We identified DEGs related to nine hormone signal transduction pathways. An overview of gene expression patterns in response to graphene treatment in maize roots is illustrated in Fig 6. Four auxin-responsive genes, such as gene-LOC100281448 (IAA9), gene-LOC100191976 (auxin-binding protein ABP20 precursor), gene-LOC103642166 (auxin response factor 11), and gene-PIN5c (auxin efflux carrier PIN5c) were differentially expressed in maize under graphene treatment conditions, indicating crosstalk between graphene and auxin signaling. This suggests that auxin modulates the plant response to graphene by altering the expression of genes involved in root growth regulation.

The CKN signaling pathway plays an important role in plant growth regulation. Four genes associated with the CKN signaling pathway exhibited significant differential expression in response to graphene treatment (Fig 6). The expression levels of gene-cko1 (cytokinin oxidase1), gene-LOC100280143 (cytokinin-N-glucosyltransferase 1), and gene-LOC100282611 (cytokinin-O-glucosyltransferase 2) were increased. We found that five genes associated with the GA pathway were also upregulated, including gene-LOC100283080 (Gibberellin 20 oxidase 2), gene-LOC100283652 (gibberellin receptor GID1L2), and gene-gar1 (gibberellin responsive 1). JA and SA play important roles in plant defense responses. The expression of six JA-related genes, including gene-LOC100283794 (jasmonate-induced protein), gene-LOC103629478 (jasmonate O-methyltransferase) and gene-LOC100273620 (jasmonate-regulated gene 21), and one SA-related gene (salicylic acid-binding protein 2) were upregulated in the roots of maize subjected to graphene treatment. Two genes involved in brassinosteroid (BR) and strigolactone (SL) signal transduction were induced in response to graphene treatment, suggesting the roles of emerging functions of graphene-responsive hormones. Together, these results demonstrate that hormones might form a complex regulatory network related to the graphene response in the roots.

### Nitrogen and potassium metabolism

**Fig 7:**
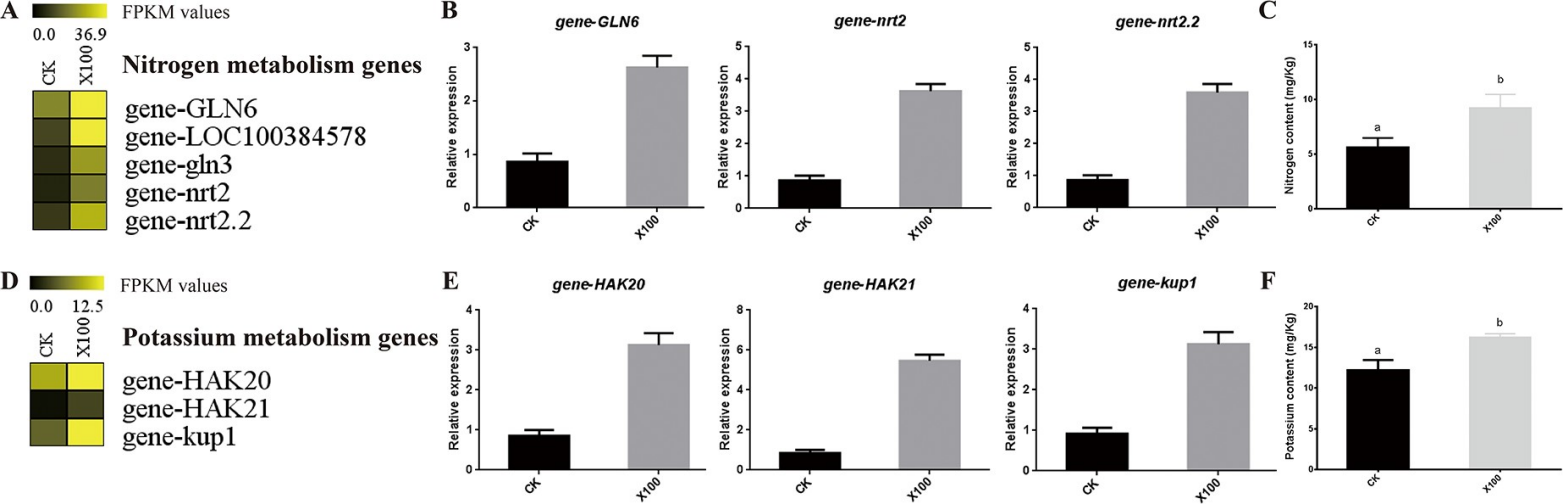
Upregulated expression of genes involved in nitrogen and potassium metabolism. (A) Nitrogen metabolism gene expression profile based on RNA-seq data. (B) Validation of the nitrogen metabolism gene expression by qRT-RCR. (C) Nitrogen content in the rhizosphere soil of maize seedlings in the CK and 50 mg/L graphene treatment. Values indicate mean ± SE (n = 5). *p* < 0.05 obtained by the Student’s *t*-test. (D) Expression profile of potassium metabolism genes based on RNA-seq data. (E) Validation of the potassium metabolism gene expression by qRT-RCR. (F) Potassium content in the rhizosphere soil of maize seedlings in the CK and 50 mg/L graphene treatment. Values indicate mean ± SE (n = 5). *p* < 0.05 obtained by the Student’s *t*-test.

We also identified eight nitrogen and potassium metabolism genes that were differentially expressed (Fig 7). All five nitrogen metabolism genes were upregulated (Fig 7A) and three (gene-GLN6, gene-nrt2, and gene-nrt2.2) were validated by qRT-PCR analysis (Fig 7B). These genes were annotated as the glutamine synthetase root isozyme 1 and ammonium transporter, which are involved in nitrogen transmembrane transport and root development. Consequently, we measured NH_4_^+^ content in the seedling rhizosphere soil, which was significantly increased by up to 1.64 times in response to the 50 mg/L graphene treatment (Fig 7C).

Expression levels of three potassium metabolism genes (gene-HAK20, gene-HAK21, and gene-kup1) were also upregulated based on RNA-seq (Fig 7D) and qRT-PCR data (Fig 7E). These genes are involved in potassium ion transmembrane transport and uptake; therefore, we also measured the K^+^ content in the seedling rhizosphere soil, finding an increase of 1.33 fold in response to the 50 mg/L graphene treatment (Fig 7F). These results indicate that parameters associated with soil fertility, such as the content of NH_4_^+^ and K^+^, may be elevated after irrigation with graphene to further promote the growth and development of maize seedling roots.

### qRT-PCR identification of DEGs

To validate the RNA-seq results, 20 DEGs were screened by qRT-PCR, including fourteen TF, three nitrogen metabolism, and three potassium metabolism genes. We analyzed the expression of these genes using quantitative real-time PCR (qRT-PCR) and compared the results with the RNA-seq data (Table 2). These transcripts exhibited similar expression patterns in the qRT-PCR and RNA-seq experiments and the correlation coefficient between the two sets of data was 0.7783 (Table 2).

**Table 2:** qRT-PCR validation of the RNA-seq results.

Gene ID	Function annotations	RNA-seq		qRT-PCR		Regulation
		Fold change	FDR	Fold change	*p*-value	
gene-EREB60	AP2-EREBP transcription factor	2.74	6.12E-04	2.28	2.16E-03	up
gene-LOC541743	Transcription factor MYB30 isoform X1	3.51	4.87E-03	5.58	2.67E-06	up
gene-MYB64	Transcription repressor MYB6	2.48	1.42E-06	5.32	3.12E-03	up
gene-myb8	Transcription factor MYB8	3.83	5.45E-12	6.26	2.66E-02	up
gene-LOC103651266	WRKY transcription factor 51	3.74	2.39E-05	2.18	1.89E-03	up
gene-WRKY45	WRKY DNA-binding domain superfamily protein	0.37	3.91E-04	0.25	3.06E-04	down
gene-LOC103628959	MADS-box transcription factor 26	0.30	1.75E-04	0.38	8.46E-02	down
gene-bHLH94	bHLH DNA-binding domain superfamily protein	2.33	1.88E-04	4.37	4.56E-03	up
gene-LOC103631852	Transcription factor EMB1444	2.22	4.93E-04	3.89	2.49E-05	up
gene-LOC103633674	NAC transcription factor 32	8.55	2.85E-04	6.26	1.80E-03	up
gene-LOC103653847	NAC domain-containing protein 7	0.20	6.75E-03	0.13	5.17E-03	down
gene-LOC103625838	Transcription factor JUNGBRUNNEN 1	2.46	3.63E-03	3.36	4.39E-02	up
gene-LOC103630375	Ethylene-responsive transcription factor ERF020	0.36	9.69E-03	0.29	3.68E-05	down
gene-LOC103635988	Ethylene-responsive transcription factor WRI1	2.86	2.45E-04	4.16	6.44E-03	up
gene-GLN6	Glutamine synthetase root isozyme 1	2.88	2.34E-07	2.65	3.32E-02	up
gene-nrt2	Ammonium transport 2	3.42	3.66E-03	3.56	1.26E-04	up
gene-nrt2.2	High affinity ammonium transporter	3.14	7.32E-04	3.68	1.86E-03	up
gene-HAK20	Potassium transporter 5	3.00	8.11E-03	3.22	3.36E-03	up
gene-HAK21	Potassium transporter 21-like isoform X1	4.87	4.65E-15	5.56	3.72E-03	up
gene-kup1	Potassium ion uptake permease 1	3.06	9.09E-09	3.26	4.70E-03	up

## Discussion

Recently, research on carbon nanomaterials has focused on applications in agriculture and forestry [6, 12, 58–60]. Studies have shown that graphene carbon nanotubes may affect the growth of maize roots, promote the growth of seminal roots, and exert no effect on the growth of primary roots, but may restrain the growth of root hairs [58]. Liu et al. [12] showed that graphene could promote rice seed germination, and could affect root development and other physiological indicators. Graphene could interact with plants through root irrigation or leaf spraying. Graphene-induced plant growth occurs at low concentrations, but demonstrates inhibitory effects on plant growth at high concentrations. However, the mechanism of graphene interaction with plants has not been thoroughly characterized. In this study, we determined the transcriptomic response of maize roots after exposure to 50 mg/L graphene, which is considered a low-concentration solution.

Using RNA-seq, we successfully identified 962 DEGs in the roots of *Z*. *mays* subjected to graphene treatment. After exposure to graphene, the number of upregulated DEGs was higher than the number of downregulated DEGs. Functional characterization of DEGs show enrichment in the categories of transporter activity, TF activity, metabolisms and plant hormone signal transduction, response to stimulus, and detoxification.

Previous research suggests that plant responses to graphene include altered expression of TF genes, as seen by the several WRKY genes induced by graphene in the roots of treated maize. Importantly, WRKY TFs often act as activators or repressors to modulate important plant processes [61]. For example, Li et al. [39] showed that WRKY genes were involved in the root elongation in *Arabidopsis*, while Wang et al. [40] reported a WRKY TF gene that could affect adventitious root formation in *Catalpa Scop*. MYB and MYB-like TF families are also important, as they are involved in controlling trichome development and root hair formation [44–47]. Additionally, the R2R3 MYBs, the basic helix-loop-helix (bHLH) factors, and the WD40 repeat (WDR) protein, play crucial roles in trichome development. These three groups of TFs form a trimeric activator complex, MYB-bHLH-WDR (MBW), which positively regulates the expression of downstream targets and, in turn, induces trichome formation [62]. Studies have also shown that bHLH TF genes are involved in root hair and meristem development in plants [41–43], and NAC TF genes regulate lateral root development in potatoes [48] and enhance root length in wheat [49]. In our study, we identified several differentially expressed TF genes that respond to graphene treatment, namely WRKY, MYB, MYB-like, bHLH, and NAC TF genes. This suggests that graphene stimulates maize root growth by upregulating the expression of TF genes.

## Conclusions

In this study, we demonstrated that low-concentration graphene treatment (50 mg/L) promotes root growth and development in *Z*. *mays*. Thereafter, we systematically characterized the influence of graphene on multiple metabolic pathways of *Z*. *mays* roots, including producing alterations in the expression of phytohormones, TF, and transporter genes. These genes are potential candidates that respond to graphene treatment by promoting root growth and development. These results provide a theoretical foundation for subsequent research detailing the molecular mechanisms underlying the interaction between graphene and maize roots.

## Supporting information

S1 Fig Phenotype of *Zea mays* seedlings with roots watering with five different concentrations of graphene and a control. (TIF)

S2 Fig Overview of maize root transcriptome response to 50 mg/L graphene (X100) and the control (CK). (A) Pearson correlation coefficient (PCC) of analysis of all genes between the six samples. (B) Principal component analysis of all samples. Red and light blue colors represent the samples of CK and those exposed to 50 mg/L graphene, respectively. (C) Volcano plot of differentially expressed genes. (D) The number of upregulated and downregulated genes.
(TIF)

S3 Fig COG database for classification of the differentially expressed gene function and homology. (TIF)

S4 Fig KEGG pathway analysis for identification of the differentially expressed gene functional categorization. (TIF)

S1 Table List of forward and reverse primers used for qRT-PCR analyses. (DOCX)

S2 Table Characteristics of the RNA-sequencing data obtained from analysis of six root samples of maize. (DOCX)

S3 Table Mapping results of RNA-seq clean reads from obtained from analysis of six root samples using the *Zea mays* genome of B73. (DOCX)
