# Supplementary figures and images for: Influence of graphene on the multiple metabolic pathways of Zea mays roots based on transcriptome analysis
Source: PLoS One. 2021 Jan 4;16(1):e0244856. doi: 10.1371/journal.pone.0244856 (PMC7781479; doi:10.1371/journal.pone.0244856)

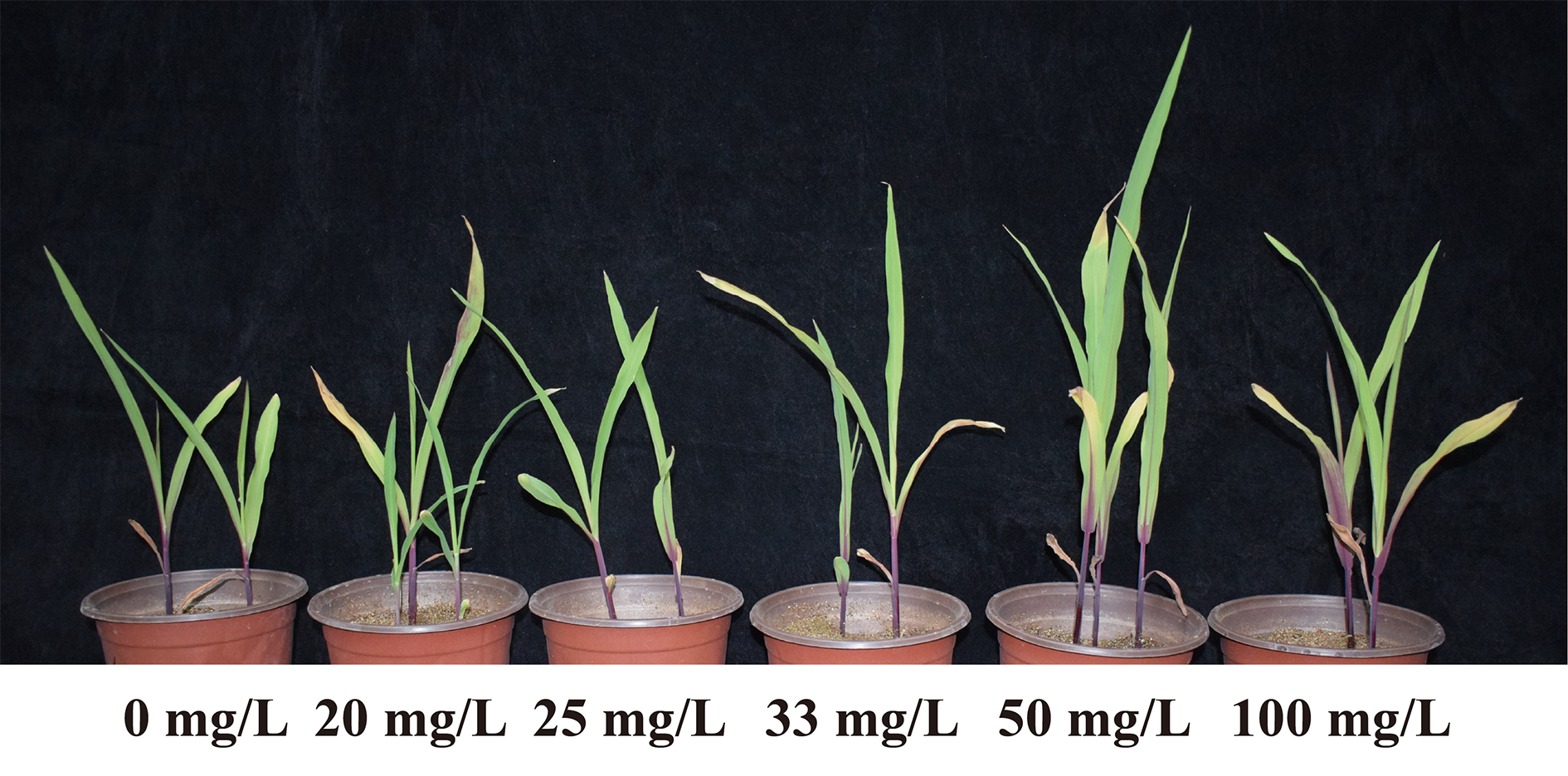

Supplement: S1 Fig — (TIF) [file pone.0244856.s001.tif]

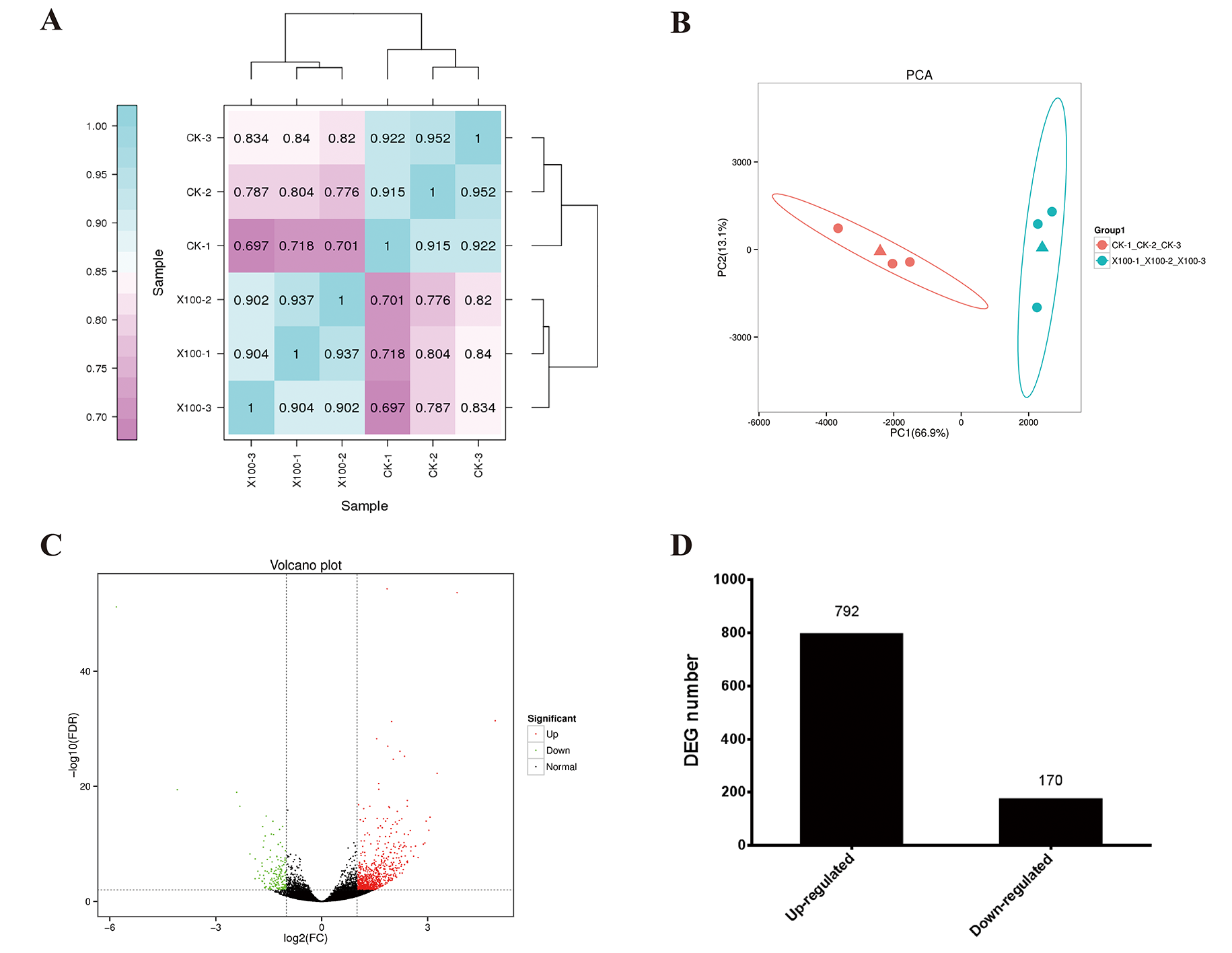

Supplement: S2 Fig — (A) Pearson correlation coefficient (PCC) of analysis of all genes between the six samples. (B) Principal component analysis of all samples. Red and light blue colors represent the samples of CK and those exposed to 50 mg/L graphene, respectively. (C) Volcano plot of differentially expressed genes. (D) The number of upregulated and downregulated genes. (TIF) [file pone.0244856.s002.tif]

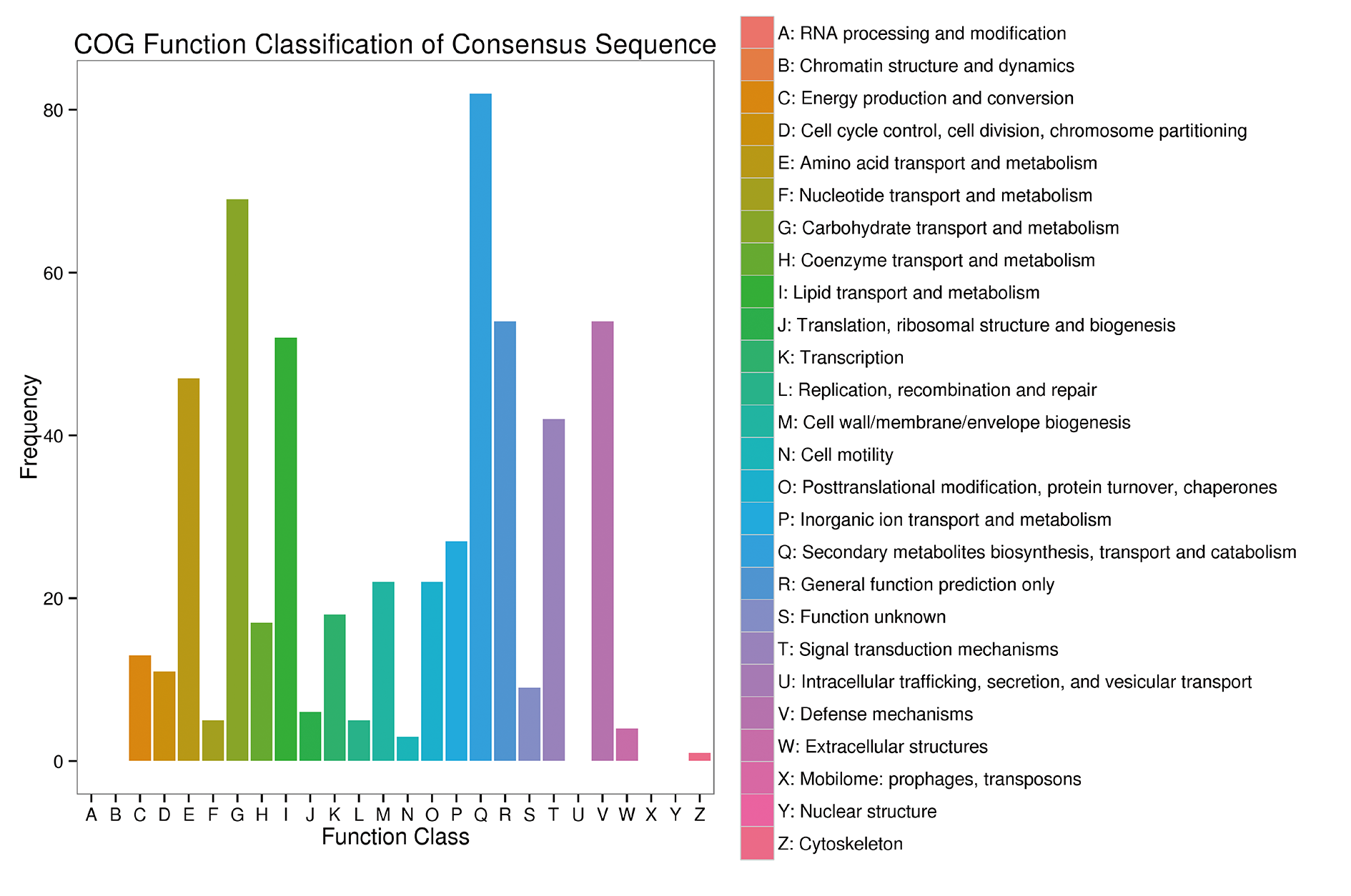

Supplement: S3 Fig — (TIF) [file pone.0244856.s003.tif]

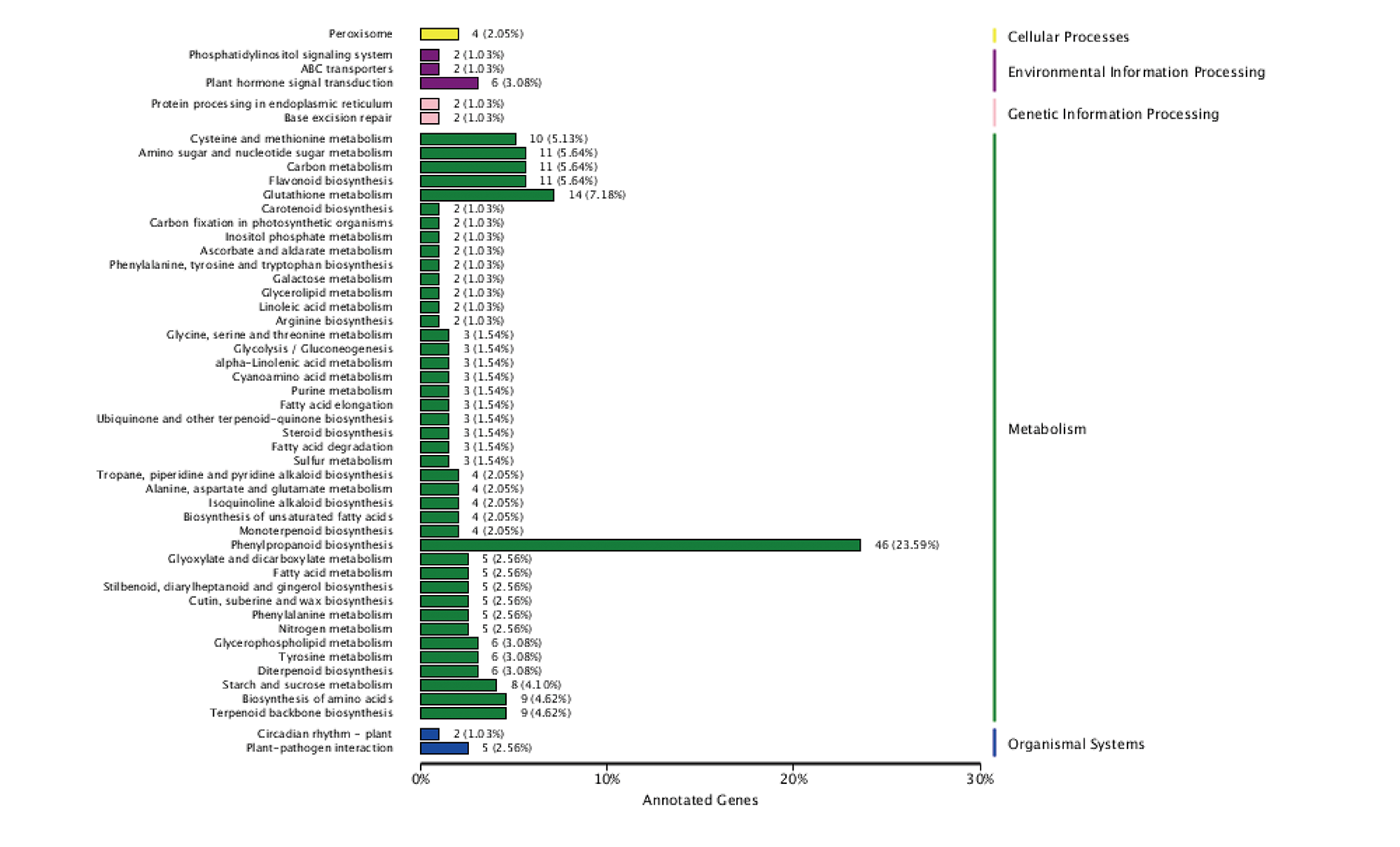

Supplement: S4 Fig — (TIF) [file pone.0244856.s004.tif]
