# Supplementary material for: Influence of graphene on the multiple metabolic pathways of Zea mays roots based on transcriptome analysis
Source: PLoS One. 2021 Jan 4;16(1):e0244856. doi: 10.1371/journal.pone.0244856 (PMC7781479; doi:10.1371/journal.pone.0244856)
Supplement: S1 Table — (DOCX) [file pone.0244856.s005.docx]

**S1 Table.** List of forward and reverse primers used for qRT-PCR analyses.

| Gene Name | Forward Primer (5'to3') | Reverse Primer (5'to3') |
| --- | --- | --- |
| gene-EREB60 | TATAGGGAGGGGTTTGGCCT | CGGACATGGTGTCGTCTGTT |
| gene-LOC541743 | GCATTCCGTGATTCTGTCGC | GTTTCAGCCCTGCTTTCTGC |
| gene-MYB64 | CTACTGGAACACGCACATCAAG | CTACTGGAACACGCACATCAAG |
| gene-myb8 | AAGCAGCTTTCGCCACCAT | TCCGCTAGCTACAACTCCCT |
| gene-LOC103651266 | AATTGCGTTCCGAGTGAGGT | CGCTCTCTGATCTGCTCGTT |
| gene-WRKY45 | ATGGGAGGAGCAGTAGAGCA | ACTCCACCTTCTTCTTGGCG |
| gene-LOC103628959 | CCGGTGTCTGTCGATCTGTG | TGGTTTCTTGCTTTGCGTCC |
| gene-bHLH94 | AGCTTCCTCTTGGACACTGC | GCTCCCTCACGAACACGATT |
| gene-LOC103631852 | ACGTCTCTTCCGTCCGTTTC | GGCCGCGGCTTTGTAATTT |
| gene-LOC103633674 | GGGACGAGTACACCAACGG | TACTAAGCACGCCGTGATGG |
| gene-LOC103653847 | CCTCTCATCCTGGCGTTTGT | CAGATCGAGTCCACCAGTCG |
| gene-LOC103625838 | ACGGTAGGTGGAGAGAAGGA | ACCTCTACAGCTTCTTGCGT |
| gene-LOC103630375 | GGGAAGTGGGTGTCGGAGAT | AATAATGCCAGCGAGAGCGG |
| gene-LOC103635988 | GGAGGAAGAGCAGTGGCTTT | GCCACGTGATGTAGGACCTC |
| gene-GLN6 | GCAAAGCAAGGGTGATGTGT | ACTGTCCAGGCATGACTTCG |
| gene-nrt2 | TACTACGCCTCCGAGTGGAA | CGCGCGCACAGTAATAACAA |
| gene-nrt2.2 | TACTACGCCTCCGAGTGGAA | CGCGTTACAAGGCATACACG |
| gene-HAK20 | GTTTGGTACGCGTTGATGCT | TCGTCTCCGAATGCTGGATG |
| gene-HAK21 | TGTTCGCTGATCTTGGGCAT | CCAACGACTGTGGCTAGTGT |
| gene-kup1 | ACTAGGAACCATCCCTCCCC | GAGCAAGAAACCGCAAAGCA |
| ZmGAPDH | CTGGTTTCTACCGACTTCCTTG | CGGCATACACAAGCAGCAAC |
