# Supplementary material for: Influence of graphene on the multiple metabolic pathways of Zea mays roots based on transcriptome analysis
Source: PLoS One. 2021 Jan 4;16(1):e0244856. doi: 10.1371/journal.pone.0244856 (PMC7781479; doi:10.1371/journal.pone.0244856)
Supplement: S2 Table — (DOCX) [file pone.0244856.s006.docx]

S2 Table. Characteristics of the RNA-sequencing data obtained from analysis of six root samples of maize.

| Samples | Clean reads | Clean bases | GC Content | %≥Q30 |
| --- | --- | --- | --- | --- |
| CK-1 | 21,833,927 | 6,534,423,536 | 55.45% | 93.20% |
| CK-2 | 28,787,785 | 8,616,729,562 | 54.99% | 93.40% |
| CK-3 | 30,063,926 | 9,002,452,888 | 55.78% | 93.25% |
| X100-1 | 29,702,272 | 8,890,781,116 | 55.68% | 93.13% |
| X100-2 | 25,463,482 | 7,611,853,658 | 56.68% | 93.72% |
| X100-3 | 24,781,394 | 7,416,597,484 | 55.70% | 92.92% |

Note: X100 represents root samples treated with 50 mg/L graphene.
