# Supplementary material for: Influence of graphene on the multiple metabolic pathways of Zea mays roots based on transcriptome analysis
Source: PLoS One. 2021 Jan 4;16(1):e0244856. doi: 10.1371/journal.pone.0244856 (PMC7781479; doi:10.1371/journal.pone.0244856)
Supplement: S3 Table — (DOCX) [file pone.0244856.s007.docx]

S3 Table. Mapping results of RNA-seq clean reads from obtained from analysis of six root samples using the *Zea mays* genome of B73.

| Sample ID | Total Reads | Mapped Reads | Unique Mapped Reads | Multiple Mapped Reads | Reads Map to “+” | Reads Map to “-” |
| --- | --- | --- | --- | --- | --- | --- |
| CK-1 | 43,667,854 | 38,122,167 (87.30%) | 37,155,829 (85.09%) | 966,338 (2.21%) | 18,940,361 (43.37%) | 18,995,091 (43.50%) |
| CK-2 | 57,575,570 | 45,860,276 (79.65%) | 44,714,357 (77.66%) | 1,145,919 (1.99%) | 22,779,899 (39.57%) | 22,862,344 (39.71%) |
| CK-3 | 60,127,852 | 52,000,249 (86.48%) | 50,634,750 (84.21%) | 1,365,499 (2.27%) | 25,827,531 (42.95%) | 25,909,932 (43.09%) |
| X100-1 | 59,404,544 | 50,093,858 (84.33%) | 48,742,517 (82.05%) | 1,351,341 (2.27%) | 24,895,678  (41.91%) | 24,973,928 (42.04%) |
| X100-2 | 50,926,964 | 39,635,692 (77.83%) | 38,530,237 (75.66%) | 1,105,455 (2.17%) | 19,686,355 (38.66%) | 19,763,994 (38.81%) |
| X100-3 | 49,562,788 | 41,862,837 (84.46%) | 40,732,380 (82.18%) | 1,130,457 (2.28%) | 20,797,383 (41.96%) | 20,860,121 (42.09%) |

Note: X100 represents root samples treated with 50 mg/L graphene.
